# A Comparison of Post-marketing Measures Imposed by Regulatory Agencies to Confirm the Tissue-Agnostic Approach

**DOI:** 10.3389/fmed.2022.893400

**Published:** 2022-06-14

**Authors:** Jorn Mulder, Odile C. van Stuijvenberg, Paula B. van Hennik, Emile E. Voest, Anna M. G. Pasmooij, Violeta Stoyanova-Beninska, Anthonius de Boer

**Affiliations:** ^1^Dutch Medicines Evaluation Board, Utrecht, Netherlands; ^2^The Netherlands Cancer Institute, Amsterdam, Netherlands; ^3^Oncode Institute, Amsterdam, Netherlands; ^4^Utrecht Institute for Pharmaceutical Sciences, Utrecht University, Utrecht, Netherlands

**Keywords:** FDA, EMA, PMDA, tissue-agnostic indication, post-marketing measures

## Abstract

There are currently four anti-cancer medicinal products approved for a tissue-agnostic indication. This is an indication based on a common biological characteristic rather than the tissue of origin. To date, the regulatory experience with tissue-agnostic approvals is limited. Therefore, we compared decision-making aspects of the first tissue-agnostic approvals between the Food and Drug Administration (FDA), European Medicines Agency (EMA) and Pharmaceuticals and Medical Devices Agency (PMDA). Post-marketing measures (PMMs) related to the tissue-agnostic indication were of specific interest. The main data source was the publicly available review documents. The following data were collected: submission date, approval date, clinical trials and datasets, and PMMs. At the time of data collection, the FDA and PMDA approved pembrolizumab, larotrectinib, and entrectinib for a tissue-agnostic indication, while the EMA approved larotrectinib and entrectinib for a tissue-agnostic indication. There were differences in analysis sets (integrated vs. non-integrated), submission dates and requests for data updates between agencies. All agencies had outstanding issues that needed to be addressed in the post-market setting. For pembrolizumab, larotrectinib and entrectinib, the number of imposed PMMs varied between one and eight, with the FDA requesting the most PMMs compared to the other two agencies. All agencies requested at least one PMM per approval to address the remaining uncertainties related to the tissue-agnostic indication. The FDA and EMA requested data from ongoing and proposed trials, while the PMDA requested data from use-result surveys. Confirmation of benefit in the post-marketing setting is an important aspect of tissue-agnostic approvals, regardless of agency. Nonetheless, each approach to confirm benefit has its inherent limitations. Post-marketing data will be essential for the regulatory and clinical decisions-making of medicinal products with a tissue-agnostic indication.

## Introduction

On May 23, 2017, the Food and Drug Administration (FDA) approved for the first time a medicinal product for a tissue-agnostic indication, i.e., an indication based on a common biological characteristic rather than the tissue of origin (1). This approval concerned pembrolizumab for the treatment of patients with microsatellite instability-high (MSI-H) or mismatch repair deficient (dMMR) tumors. During the following years, the FDA approved three other medicinal products for a tissue-agnostic indication and pembrolizumab for an additional tissue-agnostic indication (2–5). The European Medicines Agency (EMA) and Pharmaceuticals and Medical Devices Agency (PMDA), as well as other agencies, also recommended approval for medicinal products for a tissue-agnostic indication. These recommendations resulted in approvals granted by the European Commission (EC) and the Japanese Ministry of Health, Labor and Welfare (MHLW), respectively.

Pembrolizumab for the treatment of MSI-H/dMMR solid tumors was granted ‘accelerated approval’ by the FDA (1). Accelerated approval is based on an effect on a surrogate endpoint that is likely to predict clinical benefit (6). For this type of approval, the drug developer is obligated to provide additional data in the post-marketing setting to confirm clinical benefit. Hence, confirmatory data will be expected for pembrolizumab for the abovementioned indication. To accommodate similar cases, the EMA also established – years ago – an expeditated approval pathway comparable to that of the FDA called “conditional marketing authorization.” Conditional marketing authorization is based on less comprehensive data than normally required (7). Importantly, both expedited programs are developed to address an unmet medical need (6, 7).

As there are currently few tissue-agnostic approvals, regulatory experience is limited. The EMA and PMDA have issued guidance documents that include information on master protocol designs and the registration of medicinal products for tissue-agnostic indications (8, 9). Another relevant document is the FDA guidance for industry on developing targeted therapies in low-frequency molecular subsets of a disease (10). However, detailed information on data requirements for tissue-agnostic approvals or how to confirm the tissue-agnostic indication in the post-marketing setting is currently not available. Therefore, we believe that evaluation of the first tissue-agnostic approvals will allow to better define the requirements for this type of indication.

The main purpose of our study was to compare decision-making aspects of the first tissue-agnostic approvals between regulatory agencies. We were specifically interested in the post-marketing measures (PMMs) imposed to resolve outstanding issues related to the tissue-angostic indication, and whether these PMMs were different between regulatory agencies. Other aspects of interest were the datasets supporting each approval and the submission and approval dates. We limited our research to the founding regulatory members of the International Council for Harmonization of Technical Requirements for Pharmaceuticals for Human Use, i.e., the FDA, EMA and PMDA.

## Methods

### Data Sources

The primary data source was the publicly available review documents. These included the Multi-Discipline Reviews from the FDA, the European Public Assessment Reports (EPARs) from the EMA, and the Review Reports from the PMDA. Other documents used in this study were the Approval Letters from the FDA, Summary of Product Characteristics (SmPCs) from the EMA, and the List of Approved Products from the PMDA. Review documents are available at the website of each respective agency. Review documents are available at: https://www.ema.europa.eu/en/medicines, https://www.accessdata.fda.gov/scripts/cder/daf/, and https://www.pmda.go.jp/english/review-services/reviews/approved-information/drugs/0001.html.

### Data Collection

The following data were collected from the review documents: submission date, approval date, (pivotal) clinical trials, clinical datasets, and PMMs. For procedures reviewed by the EMA and the PMDA, the approval date was the date the EC and MHLW granted approval, respectively. The clinical datasets were those initially included in the submission, i.e., without any updates. For the post-marketing activities the overarching term “post-marketing measures” was used. This includes the “postmarked requirements,” “post-authorization measures” and “post-marketing investigations” as defined by the FDA, EMA and PMDA, respectively. The final date for data collections was 14 May 2021 and last check for tissue-agnostic approvals per agency was 1 October 2021.

### Data Analysis

The submission and approval dates, clinical data packages, and the PMMs were descriptively compared between agencies. No statistics were used in this study.

## Results

### Approvals per Regulatory Agency

**Figure 1:**
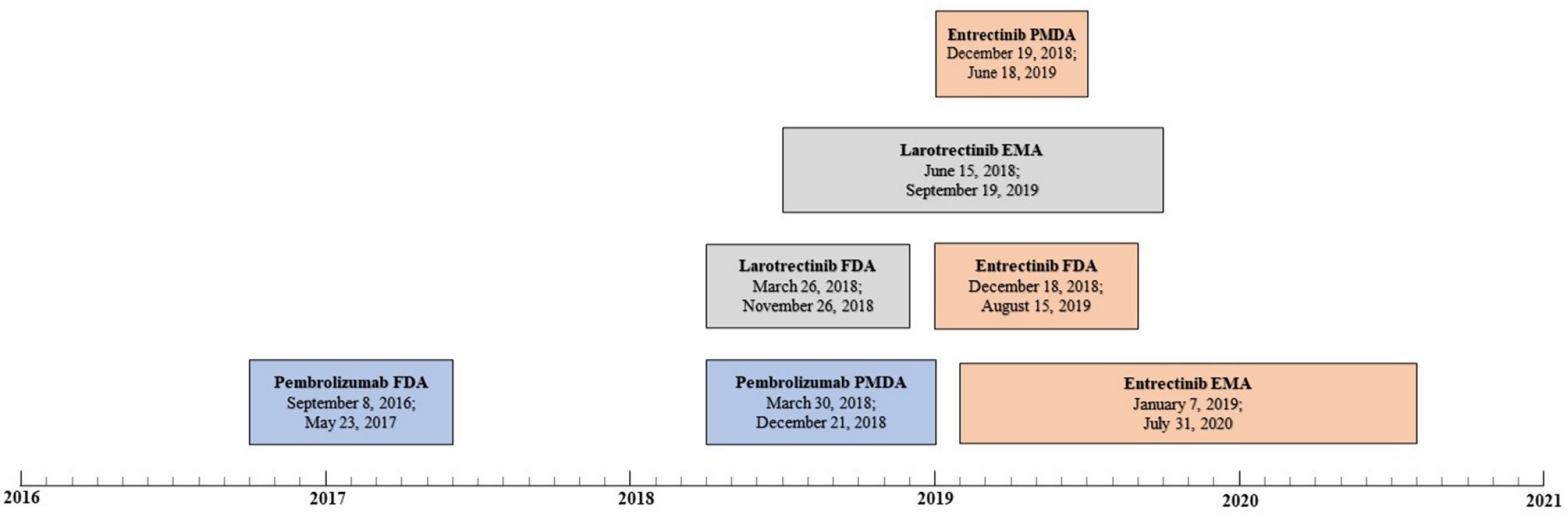
Review timelines for medicinal products for tissue-agnostic indications per agency (pembrolizumab in blue, larotrectinib in gray, and entrectinib in orange). For each review, the submission date and approval data are displayed, respectively.

Both the FDA and MHLW approved pembrolizumab for the treatment of patients with MSI-H or dMMR solid tumors (Figure 1). The application was first submitted to the FDA, with a difference of 571 days between submissions. The FDA granted “accelerated approval” and the MHLW granted “partial change approval.” Of note, on July, 2021 an extension of indication was applied for at the EMA for pembrolizumab as monotherapy in the treatment of unresectable or metastatic MSI-H or dMMR colorectal, endometrial, gastric, small intestine, biliary, or pancreatic cancer in adults who have received prior therapy (11). The review was ongoing at the final check for data availability (October 1, 2021). The FDA also approved pembrolizumab for the treatment of patients with Tumor Mutational Burden (TMB)-High solid tumors, i.e., a second tissue-agnostic indication for this medicinal product (12). However, no review documents were available for this approval at the final check for data availability.

The FDA and EC approved larotrectinib for the treatment of solid tumors that have a neurotrophic tyrosine receptor kinase (NTRK) gene fusion (Figure 1). The application was first submitted to the FDA, and the difference between submissions was 82 days. The FDA granted an “accelerated approval,” and the EC granted a “conditional marketing authorization.” On March 23, 2021, the MHLW approved larotrectinib, but no review documents were available at the final check for data availability (13).

The FDA, EC and MHLW approved entrectinib for the treatment of solid tumors that have a NTRK gene fusion (Figure 1). The application was first submitted to the FDA but the difference between the submission dates was small – 21 days or less. The FDA granted an “accelerated approval,” the EC granted a “conditional marketing authorization” and the MHLW granted a “new approval.”

Finally, on August 17, 2021, the FDA granted an “accelerated approval” for dostarlimab for the treatment of patients with dMMR recurrent or advanced solid tumors. Since only the FDA approved dostarlimab for a tissue-agnostic indication no comparison could be made between the three agencies. Therefore, dostarlimab was not included in our analysis.

### Clinical Data Supporting the Submission

The FDA and PMDA approved pembrolizumab for MSI-H/dMMR solid tumors based on five and two clinical trials, respectively (Table 1). Data from KEYNOTE158 and KEYNOTE164 were submitted to both agencies, but different data cut-off dates were used. The most frequent tumor types included in the efficacy datasets were colorectal cancer and endometrial cancer (Supplementary Table 1). The application submitted to the FDA included pooled efficacy data from five clinical trials and pooled safety data from two trials, i.e., KEYNOTE016A and KEYNOTE164. The pooled efficacy population consisted of 149 patients. The primary endpoint was overall response rate (ORR). ORR was 35.6% (95% CI: 27.9, 43.8). The pooled safety population consisted of 89 patients. During the review, the FDA received updated efficacy data from KEYNOTE158 and KEYNOTE164. The data cut-off dates for the updated efficacy data were August 7, 2016 and November 23, 2016 for KEYNOTE158 and August 3, 2016 and November 23, 2016 for KEYNOTE164. Updated safety data was also provided, but the safety review was based on the initial dataset. The application submitted to the PMDA included separately presented efficacy and safety data from KEYNOTE158 and KEYNOTE164. ORR was the primary endpoint in both studies. Response rates were 34.9% (95% CI: 24.8, 46.2) and 27.9% (95% CI: 17.1, 40.8), respectively.

**Table 1:** Clinical trials supporting the approval of pembrolizumab for the treatment of MSI-H/dMMR tumors.

					**FDA**		**PMDA**	
**Name**	**Design**	**Population**	**Dose**	**Primary endpoint**	**Number of patients**	**Data cut-off date**	**Number of patients**	**Data cut-off date**
KEYNOTE164	Open-label, multicenter, Phase 2 trial	Previously treated patients with MSI-H CRC	200 mg every 3 weeks	ORR	61	June 3, 2016	61	February 10. 2017
KEYNOTE016A	Investigator-initiated, open label, multi-center, 2-stage, Phase 2 trial	Previously treated patients with MSI-H CRC	10 mg/kg every 2 weeks	ORR	28	February 19, 2016	N.A.	N.A.
KEYNOTE016C	Investigator-initiated, open label, multi-center, 2-stage, Phase 2 trial	Previously treated patient with MSI-H non-CRC	10 mg/kg every 2 weeks	ORR	30	April 13, 2016	N.A.	N.A.
KEYNOTE158	Open-label, multicenter, multi-cohort, Phase 2 trial	Patients with advanced solid tumors evaluated for predictive biomarkers	200 mg every 3 weeks	ORR	19	June 17, 2016	94	April 28, 2017
KEYNOTE012	Multicenter, multi-cohort trial	Patients with advanced solid tumors	10 mg/kg every 2 weeks	ORR	6	April 26, 2016	N.A.	N.A.
KEYNOTE028	Open-label, multicenter, multi-cohort, Phase 1b trial	Patients with PD-L1 positive advanced solid tumors	10 mg/kg every 2 weeks	ORR	5	June 20, 2016	N.A.	N.A.

*CRC, colorectal cancer, FDA, Food and Drug Administration; MSI-H, microsatellite instability-high; N.A., not applicable; ORR, overall response rate; PD-L1, programmed cell death-1 ligand 1; PMDA, Pharmaceuticals and Medical Devices Agency*.

The FDA and EMA approved larotrectinib for NTRK fusion-positive solid tumors on the basis of three clinical trials (Table 2). The most frequent tumor types included in the efficacy datasets were sarcoma and salivary gland (Supplementary Table 1). Both applications included pooled efficacy and safety data from the three clinical trials, but there were small differences in data cut-off dates and population sizes. The primary endpoint of the integrated analysis was ORR. The agencies focused their review on different analysis sets, which resulted in differently sized efficacy populations. The FDA focused on the primary analysis set (PAS), which consisted of the first 55 patients with NTRK fusion-positive solid tumors enrolled across the three studies. ORR was 75% (95% CI: 61, 85). The pooled safety population consisted of 144 patients from three studies. During the review, the FDA received updated efficacy and safety data. The updated clinical data cut-off date was February 19, 2018. The EMA initially focused their review on the PAS – same as the FDA – but considered the second extended PAS (ePAS2) to be the main analysis set. This analysis set consisted of 93 patients from the same clinical trials but was based on updated efficacy data. ORR in the ePAS2 was 72% (95% CI: 62, 81). The pooled safety dataset consisted of 176 patients from three studies During the review, the EMA also received updated safety data. The updated clinical cut-off date was July 30, 2018.

**Table 2:** Clinical trials supporting the approval of larotrectinib for the treatment of NTRK tumors.

					**FDA**		**EMA**	
**Name**	**Design**	**Population**	**Dose**	**Primary endpoint**	**Number of patients**	**Data cut-off date**	**Number of patients**	**Data cut-off date**
LOXO-TRK-14001	Open-label, multicenter, Phase 1 Study	Adult patients with solid tumors	50–400 mg/day QD or BID	Safety, MTD, RP2D	66	July 17, 2017	70	February 19, 2018
LOXO-TRK-15002	Open-label, multicenter, Phase 2 Basket Study	Patients 12 years of age or older with NTRK fusion advanced cancer	100 mg BID	BOR	47	July 17, 2017	63	February 19, 2018
LOXO-TRK-15003	Open-label, Multicenter, Phase 1/2 Study	Pediatric patients with advanced solid or primary central nervous system tumors	Dosing based on adult equivalent of 100 or 150 mg BID, then 100 mg/m^2^ BID (maximum of 100 mg BID)	Safety, DLT (part 1); ORR (part 2)	31	July 17, 2017	43	February 19, 2018

*BID, twice daily; BOR, best overall response; EMA, European Medicines Agency; FDA, Food and Drug Administration; MTD, maximum tolerated dose; NTRK, Neurotrophic Tyrosine Receptor Kinase; ORR, overall response rate; QD, once daily; RP2D, recommended phase 2 dose*.

The FDA and EMA approved entrectinib for NTRK fusion-positive solid tumors on the basis of four clinical trials, while the PMDA approved entrectinib for NTRK fusion-positive solid tumors on the basis of three clinical trials (Table 3). The most frequent tumor types included in the efficacy datasets were sarcoma and salivary gland (Supplementary Table 1). The applications submitted to the FDA and EMA included pooled efficacy and safety data from these clinical trials. The primary endpoint for the integrated efficacy analysis was ORR. The FDA and EMA focused on different analysis sets, which resulted in differently sized efficacy populations. The FDA focused on the PAS, which consisted of the first 54 patients enrolled in ALKA-372-001, RXDX-101-01, and RXDX-101-02. ORR was 57.4% (95% CI: 43.2, 70.8). The pooled safety population consisted of 355 patients from four studies. Updated efficacy and safety data was submitted. The updated clinical data cut-off date was October 31, 2018. The EMA initially focused on the PAS, but considered the extended PAS to be the main analysis set. This analysis set consisted of 74 patients from ALKA-372-001, RXDX-101-01, and RXDX-101-02, but was based on updated efficacy data. ORR in the extended PAS was 63.5% (95% CI: 51.5, 74.4). The pooled safety population consisted of 355 patients from four studies. During the review, the EMA also received updated safety data. The updated clinical data cut-off date was Oct 31, 2018. The application submitted to the PMDA included efficacy data from RXDX-101-02 and separately presented safety data from ALKA-372-001, RXDX-101-01, and RXDX-101-02.The PMDA focused on the PAS that consisted of 51 patients from study RXDX-101-02. The primary endpoint was ORR. Response rate was 56.9% (95% CI: 42.3, 70.7).

**Table 3:** Clinical trials supporting the approval of entrectinib for the treatment of NTRK tumors.

					**FDA**	**PMDA**	**EMA**	
**Name**	**Design**	**Population**	**Primary endpoint**	**Dose**	**Number of patients**			**Data cut-off date**
ALKA-371-001	A dose escalation, Phase 1 study	Adult patients With Advanced/ Metastatic Solid Tumors	First cycle DLTs, MTD	100–1,600 mg QD	57	57	57	May 31, 2018
RXDX-101-01	Open-label, multicenter, Phase 1 study	Adult patients with Locally Advanced or Metastatic Cancer	Dose escalation part: first cycle DLTs, MTD, and a biologically effective and RP2D Dose expansion part: ORR	100–800 mg QD	76	76	76	May 31, 2018
RXDX-101-02	Open-label, multicenter, global Phase 2 Basket Study	Patients with Locally Advanced or Metastatic Solid Tumors that Harbor NTRK1/2/3, ROS1, or ALK Gene Rearrangements	ORR	600 mg QD	206	206	206	May 31, 2018
RXDX-101-03	Open-label, dose-escalation and expansion, Phase 1/1b study	Children and Adolescents with Recurrent or Refractory Solid Tumors and Primary CNS Tumors, with or without TRK, ROS1, or ALK Fusions	MTD, RP2D	Dosing nomogram based on BSA, ranging from 250 mg/m^2^ to 750 mg/m^2^	16	N.A.	16	May 31, 2018

*ALK, Anaplastic Lymphoma Kinase; BSA, body surface area; EMA, European Medicines Agency; FDA, Food and Drug Administration; MTD, maximum tolerated dose; N.A., not applicable; NTRK, Neurotrophic Tyrosine Receptor Kinase; ORR, overall response rate; QD, once daily; PMDA, Pharmaceuticals and Medical Devices Agency; ROS1, Proto-oncogen tyrosine-protein kinase 1; RP2D, recommended phase 2 dose*.

### Post-marketing Measures

The FDA imposed four PMMs and the PMDA imposed one PMM for pembrolizumab (Table 4 and Supplementary Table 2). Both agencies imposed a PMM to address the small sample size of patients with MSI-H/dMMR solid tumors other than colorectal cancer. To resolve this issue, the FDA requested the final study reports from KEYNOTE 158 and KEYNOTE 164. In contrast, the PMDA requested an use-result survey. The FDA imposed three other PMMs, which are related to safety in pediatric patients and use of companion diagnostics. The latter was not relevant to the PMDA review, as a companion diagnostic assay was approved in Japan on 10 September 2018.

**Table 4:** Post-marketing measures for pembrolizumab for the treatment of MSI-H/dMMR tumors.

**Agency**	**Type of measure**	**Short description of issue (s)**	**Short description of measure**	**Planned sample size**	**Due date**
FDA	Accelerated approval requirements	Limited experience with treatment of patients with MSI-H/dMMR tumors other than metastatic colorectal cancer and endometrial cancer	Submit the final report, including datasets, from trials conducted to verify and describe the clinical benefit of pembrolizumab in patients with MSI-H/dMMR tumors	≥124 patients (KEYNOTE164); ≥300 patients (KEYNOTE158)	March, 2023
	Postmarketing requirements under 505(o)	Limited experience of pembrolizumab in children with congenital mismatch repair deficiency syndromes	Conduct a trial to determine a reasonably safe dosage regimen in children with MSI-H/dMMR primary central nervous system malignancies	Unspecified or not applicable	March, 2023
	Postmarketing commitments subject to reporting requirements under section 506B	There remains uncertainty regarding the performance characteristics across all laboratories which may be performing tests for determination of MSI-H and dMMR tumor status	Commitment to support the availability of an *in vitro* diagnostic device that is essential to the safe and effective use of pembrolizumab for patients with dMMR tumors	Unspecified or not applicable	June, 2019
	Postmarketing commitments subject to reporting requirements under section 506B	There remains uncertainty regarding the performance characteristics across all laboratories which may be performing tests for determination of MSI-H and dMMR tumor status	Commitment to support the availability of an *in vitro* diagnostic device that is essential to the safe and effective use of pembrolizumab for patients with MSI-H tumors	Unspecified or not applicable	June, 2019
PMDA	Post marketing surveillance	Limited data is available as to the efficacy of pembrolizumab in the treatment of MSI-H tumors (except colorectal cancer)	A drug use-results survey to collect information on the characteristics of patients treated with pembrolizumab, to promptly collect data on the efficacy and safety of pembrolizumab, and to take necessary actions to ensure the proper use of pembrolizumab.	≥30 patients	Not specified

*dMMR, Mismatch repair deficient; FDA, Food and Drug Administration; MSI-H, microsatellite instability-high; PMDA, Pharmaceuticals and Medical Devices Agency*.

The FDA imposed seven PMMs and the EMA imposed three PMMs for larotrectinib. Both agencies imposed a PMM to address the small sample size of patients with NTRK fusion-positive tumors in relation to the complexity of the tissue-agnostic indication (Table 5 and Supplementary Table 2). Both agencies requested a larger dataset from (ongoing) clinical trials to resolve this issue. Unique to the PMM imposed by the EMA is that it will address two additional issues, i.e., lack of prospectively studied cohorts and secondary NTRK mutations that cause resistance to larotrectinib. In addition, both agencies imposed a PMM to address the limited safety data in pediatric patients, which resulted in comparable measures. The other PMMs were imposed by only one of the two agencies, and addressed issues related to: (1) duration of response (FDA only); (2) the dose in pediatric patients (EMA only); (3) the third dosage modification (FDA only); (4) moderate CYP inhibitors/inducers (FDA only); and (5) companion diagnostics (FDA only). The underlying issues were not identified by the other agency or were not relevant for the review. The approval of a companion diagnostic was not required by the EMA, but it is indicated in the SmPC that the presence of a NTRK gene fusion in a tumor specimen should be confirmed with a validated test. The risks related to co-administration with CYP inhibitors/inducers were addressed in the SmPC of Vitrakvi. The following statement was included: “No clinical data is available on the effect of a moderate inducer, but a decrease in larotrectinib exposure is expected.”

**Table 5:** Post-marketing measures for larotrectinib for the treatment of NTRK tumors.

**Agency**	**Type of measure**	**Short description of issue (s)**	**Short description of measure**	**Planned sample size**	**Due date**
FDA	Accelerated approval requirements	Due to the small sample size, there is an uncertainty regarding the magnitude of the treatment effect of larotrectinib in any single histologic subtype of solid tumors with an activating NTRK fusion	Submit the final report, including datasets, from ongoing and proposed trials conducted to verify and describe the clinical benefit of larotrectinib	Unspecified or not applicable	August, 2025
	Accelerated approval requirements	Not described/ The median DOR had not been reached as of the February 19, 2018 data cut-off date.	Submit the final report, including datasets, from the first 55 patients with NTRK fusion solid tumors to further characterize the duration of response in patients who achieved a complete or partial response to larotrectinib.	55 patients	March, 2020
	Postmarketing Requirements under 505 (o)	The number of pediatric patients in the safety database was small and there are inherent limitations in interpreting longitudinal growth and development information in single arm trials	Conduct a study in pediatric patients with NTRK-fusion solid tumors to evaluate the potential serious risk of adverse long-term effects of larotrectinib on the growth and development	Unspecified or not applicable	August, 2028
	Postmarketing Requirements under 505 (o)	Limited clinical experience with a third dose modification	Conduct a study in adult or pediatric patients with a body surface area of at least 1.0 m^2^ who experienced an adverse reaction requiring a third dosage modification of larotrectinib to better characterize the tolerability of this dosage modification	Unspecified or not applicable	August, 2028
	Postmarketing Requirements under 505 (o)	The effects of moderate and weak CYP3A4 inhibitors on the PK of larotrectinib have not been studied	Conduct a physiologically-based pharmacokinetic modeling study to evaluate the effect of a moderate CYP3A4 inhibitor on the pharmacokinetics of larotrectinib	Unspecified or not applicable	September, 2019
	Postmarketing commitments subject to reporting requirements under section 506B	The effects of CYP3A moderate and weak inducers on the pharmacokinetics of larotrectinib have not been studied	Conduct a physiologically-based pharmacokinetic modeling study to evaluate the effect a moderate CYP3A4 inducer on the pharmacokinetics of larotrectinib	Unspecified or not applicable	September, 2019
	Postmarketing commitments subject to reporting requirements under section 506B	Several challenges remain for NTRK fusion testing	Conduct an analytical and clinical validation study that is adequate to support labeling of an *in vitro* diagnostic device that is essential to the safe and effective use of larotrectinib	Unspecified or not applicable	July, 2021
EMA	Specific Obligations	1) The small efficacy data base raises issues with regard to the representativeness in relation to the indication sought, encompassing any solid tumor type; 2)The application is lacking in prospectively studied cohorts that could provide an unbiased estimate of ORR; and 3) Secondary NTRK mutations altering the kinase domain of TRK thus appear to be a major acquired resistance mechanism to larotrectinib	In order to further confirm the histology-independent efficacy of larotrectinib and to investigate the primary and secondary resistance mechanisms, the MAH should submit a pooled analysis for the increased sample size including the final report of study LOXO-TRK-15002	200 patients (LOXO-TRK-15002 and LOXO-TRK-15003)	June 30, 2024
	Specific Obligations	The long-term safety follow-up is limited and neurodevelopment in pediatric patients is a concern based on preclinical data.	In order to further investigate the long-term toxicity and developmental effects of larotrectinib in pediatric patients the MAH should submit the final report of study	Unspecified or not applicable	March 31, 2027
			LOXO-TRK-15003 including 5 year follow up data		
	Specific Obligations	There is uncertainty in the predicted exposure in the smallest children (< 6 years of age) due to few patients included in each age group	In order to further confirm the appropriate dose recommended in pediatric patients, the MAH should submit an updated pop PK model based on additional PK sampling in patients aged 1 month to 6 years from study LOXO-TRK-15003	Unspecified or not applicable	September 30, 2021

*CYP3A4, cytochrome P450 3A4; DOR, duration of response; EMA, European Medicines Agencies; FDA, Food and Drug Administration; PK, pharmacokinetics; MAH, marketing authorization holder; NTRK, neurotrophic tyrosine receptor kinase; ORR, overall response rate*.

The FDA imposed eight measures and the PMDA and EMA imposed two measures each for entrectinib (Table 6 and Supplementary Table 2). All three agencies imposed a PMM to address the small sample size of patients with NTRK fusion-positive tumors in relation to the complexity of a tissue-agnostic indication. The FDA and EMA both requested an expanded dataset from ongoing clinical trials. The PMDA requested an use-result survey. The agencies also imposed, sometimes integrated, PMMs to address to the risk of fractures (FDA, EMA), growth and development issues (FDA, PMDA, and EMA), and congestive heart failure (FDA, PMDA, EMA). The other PMMs were imposed by only one of the three agencies, and addressed issues related to: (1) the duration of response (FDA only); (2) off-target activity (FDA only); (3) dose in patients with hepatic impairment (FDA only); (4) companion diagnostics (FDA only); and (5) concomitant genetic mutations (EMA only). The underlying issues were not identified by the other agencies or were not relevant for the review. A companion diagnostic was already approved in Japan. The approval of a companion diagnostic was not a requirement by the EMA, but it is indicated in the SmPC that a validated assay is required for the selection of patients with NTRK gene fusion-positive solid tumors.

**Table 6:** Post-marketing measures for entrectinib for the treatment of NTRK tumors.

**Agency**	**Type of measure**	**Short description of issue (s)**	**Short description of measure**	**Planned sample size**	**Due date**
FDA	Accelerated approval requirements	Not specified	Submit the final report, including datasets, from the first 54 patients with NTRK-fusion solid tumors enrolled across ALKA-372-001, RXDX-101-01, and RXDX-101-02 to verify and describe the clinical benefit and further characterize the duration of response in patients who achieved a complete or partial response to entrectinib	54 patients	June, 2021
	Accelerated approval requirements	Due to the small sample size, there is a degree of uncertainty regarding the magnitude of the treatment effect of entrectinib in any single histologic subtype of solid tumors with an activating NTRK fusion	Submit the final report, including datasets, from ongoing and proposed trials conducted to verify and describe the clinical benefit of entrectinib	Unspecified or not applicable	March, 2027
	Postmarketing requirments under 505(o)	Entrectinib had potential activity against other receptors that could contribute to CNS effects	Determine functional activation or inhibition of off-target receptors, transporters, and/or channels that, at concentrations of 10 μM, showed >50% inhibition by entrectinib or M5 in the secondary pharmacology studies.	Unspecified or not applicable	September, 2020
	Postmarketing requirments under 505(o)	Congestive heart failure events were reported in 12 (3.4%) patients, including Grade 3 (2.3%)	Submit integrated safety analyses and supporting data from patients enrolled in clinical trial (s) designed to characterize the cardiac risks and its sequelae in patients exposed to entrectenib with reasonable precision	Unspecified or not applicable	June, 2022
	Postmarketing requirments under 505(o)	The small number of pediatric patients in the safety database, the limited duration of follow-up, and limitations inherent in interpreting longitudinal growth and development information in single arm trials	Conduct clinical trial (s) of entrectinib in pediatric patients 12 years of age and older with NTRK-fusion solid tumors to evaluate the potential serious risk of adverse long-term effects of entrectinib on growth and development	Unspecified or not applicable	August, 2029
	Postmarketing requirments under 505(o)	Seventeen (5%) adult patients and 7 (23%) pediatric patients experienced fractures	Submit integrated safety analyses and supporting data from patients enrolled in clinical trial (s) designed to characterize the risk of fractures and its sequelae in patients exposed to entrectinib with reasonable precision	Unspecified or not applicable	March, 2025
	Postmarketing requirments under 505(o)	There is no pharmacokinetic data to recommend entrectinib dose for patients with moderate and severe hepatic impairment.	Complete a pharmacokinetic trial to evaluate the effect of moderate and severe hepatic impairment on the pharmacokinetics and safety of Rozlytrek (entrectinib) compared to subjects with normal hepatic function	Unspecified or not applicable	December, 2021
	Postmarketing commitments subject to reporting requirements under section 506B	Several challenges remain for NTRK fusion testing	Commit to providing adequate analytical and clinical validation results from clinical trial data to support labeling of the F1CDx test to detect NTRK rearrangements for identifying patients who may benefit from entrectinib	Unspecified or not applicable	December, 2019
EMA	Specific Obligation	1) The estimates by tumor types are not robust due to the small sample sizes of individual subgroups and the limited number of tumor types; and 2) The available safety data in the pediatric setting is limited	In order to further confirm the histology-independent efficacy of entrectinib in adult and pediatric patients, the MAH should submit a pooled analysis for an increased sample size of NTRK fusion-positive patients from RXDX-101-02, RXDX-101-03 and any additional clinical trial conducted according to an agreed protocol	200 patients (RXDX-101-02, RXDX-101-03)	March 31, 2027
	Specific Obligation	Concomitant genetic mutations occur frequently in tumors with NTRK fusion	In order to further investigate the impact of the presence/absence of other molecular alteration on the efficacy of entrectinib, the MAH should submit the results from tumor genomic profiling by plasma and/or tissue together with clinical outcomes association per tumor histology	Unspecified or not applicable	March 31, 2027
PMDA	Post-marketing surveillance	The safety information from patients treated with entrectinib, including Japanese patients, is limited, and the number of patients with NTRK fusion-positive, advanced/recurrent solid tumors (patients eligible for entrectinib therapy) is extremely limited	to conduct a post-marketing use-results survey covering all patients treated with entrectinib, in order to obtain information on the characteristics of patients treated with entrectinib, to promptly collect data on the safety and efficacy of entrectinib, and to take necessary measures to ensure proper use of entrectinib	200 patients	Not specified
	Post-marketing surveillance	The number of pediatric patients with NTRK fusion-positive, advanced/recurrent solid tumors is extremely limited	To conduct a post-marketing use-results survey to investigate delayed growth and development in pediatric patients in clinical practice	Unspecified or not applicable	Not specified

*CNS, central nervous system; EMA, European Medicines Agencies; FD, Food and Drug Administration; F1CDx, Foundation One companion diagnostic test; MA, marketing authorization holder; NTRK, neurotrophic tyrosine receptor kinase; PMDA, Pharmaceuticals and Medical Devices Agency*.

## Discussion

This study was conducted to compare decision-making aspects of tissue-agnostic approvals between regulatory agencies. Specifically, we were interested in the PMMs imposed by the regulatory agencies to resolve outstanding issues related to the tissue-agnostic indication. At the time of our analysis, pembrolizumab, larotrectinib and entrectinib received regulatory approval for a tissue-agnostic indication(s) by most, or all three, regulatory agencies. Datasets supporting the approvals were generally small, especially for the two TRK inhibitors. Numerous PMMs were imposed by the agencies, and these were mostly imposed to address issues related to efficacy, safety, and pharmacokinetics.

There remain uncertainties concerning the treatment effect across tumor types for medicinal products for tissue-agnostic indications. Our data shows that all three agencies will receive additional data to further confirm the tissue-agnostic indication in the post-marketing setting. Larger datasets will be collected from (ongoing) clinical trials or real-world studies. These studies shall include patients with tumor types that were underrepresented in the initial datasets. However, both approaches to collect data in the post-marketing setting, i.e., clinical trials vs. real-world studies, have their inherent challenges and/or limitations. For example, clinical trials may have stringent eligibility criteria, which result in limited generalizability of the study results (14). While generalization may be improved with real-world studies, these studies are not without challenges either, which can be of operational, technical and/or methodological nature (15). For instance, the quality and completeness of information is critical to describe the patients characteristics and treatment effects (16). There is currently limited guidance available on the data requirements to confirm a tissue-agnostic indication in the post-marketing setting. In the guideline of the PMDA it is recommended that high quality data, including data from cancer types not evaluated in the clinical trials, is required after marketing authorization (9). For future applications, a global strategy toward the generation of data in the post-marketing setting may be of interest, dependent on emerging insights from the current approaches.

As the FDA and EC granted expedited approval, i.e., “accelerated approval” and “conditional marketing authorization,” respectively, the drug developers are obligated to provide confirmatory data in the post-marketing setting. Importantly, regulatory action can be taken if new data alters the benefit-risk ratio, which might also impact the tissue-agnostic indication. Randomized-controlled trials remain the gold standard to determine the efficacy and safety of a medicinal product and are often preferred as confirmatory trials. However, as addressed by the FDA reviewers, it will be challenging to conduct a randomized-controlled trial in the tissue-agnostic setting (17). Some biomarker-tumor combinations are extremely rare (18). The low prevalence of a biological characteristic may lead to recruitment challenges, even for basket trials (19). In addition, shared among medicinal products approved for a tissue-agnostic indication is the “strong scientific rationale” (20). Such a scientific/biological rationale and the demonstration of large treatment effects may bring clinical equipoise into question. This explains why confirmatory data for the tissue-agnostic approvals will be obtained from ongoing single-arm (basket) trials or real-worlds studies. Recently, Seligson el al. reflected on tissue-agnostic approvals by the FDA and suggested that real-world data can be used as an alternative strategy to confirm benefit (20). Miksad et al. described that the identification of real-world patients could expand the evidence base for the NTRK population (16). Real-world data might also provide a more comprehensive understanding of long-term effects for drugs approved on the basis of earlier phase clinical trials, i.e., phase 1 or phase 2 trials (21). More general, it has been published that post-marketing studies are sometimes delayed (22). Regardless of PMM, it is important that regulators insist that the due date for these (mandatory) measures will be met, as the confirmation of benefit is an important aspect of tissue-agnostic approvals.

The first approvals set precedent for the level of evidence necessary to register medicinal products for a tissue-agnostic indication. Our results show that tissues-agnostic approvals are supported by somewhat small (efficacy) datasets – albeit sufficient for the initial assessment of the benefit-risk balance, especially considering the complexity of a tissue-agnostic indication. Furthermore, agencies have a different approach toward reviewing the submitted data, i.e., some agencies accept integrated datasets, focus on particular analysis sets and/or request updated data. Findings from literature confirm that the size of the efficacy populations supporting the tissue-agnostic approvals can be considered smaller than generally observed for registration. For example, Tenhunen et al. reported that the median number of patients in the target population was 175 for EC approvals based on results from single-arm trials (23). This is substantially larger than the initial efficacy population supporting the approval of the TRK inhibitors. The current tissue-agnostic approvals were based on a strong biological rationale and thereby the expectation of consistency in treatment effect across tumor types (20). The importance of a strong biological rationale to support a tissue-agnostic approach is highlighted in the EMA draft guidance document. It is specified in this guidance document that the assessment of homogeneity can be conducted only if a sufficient number of patients is included in the study, which is not always feasible (8). The PMMs will allow re-assessment of homogeneity based on a larger dataset in the post-marketing setting, sometimes including data from at least 200 additional patients, based on our findings. Regarding the integrated analysis sets, Seligson et al. reported that the FDA accepted pooled data due to the consistent anti-tumor activity across trials (20). However, not every agency might accept this strategy. For instance, the PMDA considered RXDX-101-02 to be the sole pivotal study to support the application of entrectinib and focused its review on the NTRK fusion-positive cohort (24). Early dialogue between developers and regulators to discuss (clinical) development plans may prevent regulatory hurdles.

Our results show that not all developers submitted an application to all agencies at the same time. The differences in submission date for pembrolizumab are the most noticeable. For instance, only recently an extension of indication for six MSI-h tumors for pembrolizumab was submitted to the EMA (11). Remarkably, the developer did not apply for a tissue-agnostic indication in the European Union (EU) (25); a different strategy compared to the applications submitted to FDA and PMDA. Understanding why the developer did not seeking a tissue-indication is of interest, but can only be speculated on. Likewise, while the EC approved dostarlimab for the treatment of patients with dMMR/MSI-H recurrent or advanced endometrial cancer, the applicant did not apply for a tissue-agnostic indication in the EU (26). A delay in submission is one of the factors that may contribute to disparity in approved therapies between countries or continents. In the absence of regulatory approval, patients in Europe are often dependent of clinical trials or patients access programs (27). Initiatives such as the personalized reimbursement scheme incorporated in the Drug Rediscovery Protocol enable access to promising medicinal products for unregistered indications, but only on a national level (28).

To our knowledge, this is the first comparison of tissue-agnostic approvals between three independent regulatory agencies. However, our study is limited to the products that received regulatory approval for a tissue-agnostic indication(s). Additional approvals might make such a study more sensitive to determine differences in regulatory decision-making between agencies. Despite this limitation, we consider that the currently available information is sufficiently abundant to compare at least some aspects related to decision-making for tissue-agnostic approvals.

## Conclusion

The current approvals set precedent for the level of evidence necessary to register medicinal products for tissue-agnostic indication. A strong biological rationale and consistency in treatment effect across tumor types may allow for (expedited) approval, but confirmation of benefit in the post-marketing setting remains an important aspect of the tissue-agnostic approvals. There are different approaches to further confirm the tissue-agnostic indication in the post-marketing setting, albeit these are not without inherent limitations. These approaches to collect post-marketing data could complement existing data packages, but it will be of importance that “new” data are of high quality. For future applications, a global strategy toward data generation post-marketing may be of interest, dependent on emerging insights from the current approaches. Eventually, only the post-marketing data will continue to shed light on the appropriateness of the tissue-agnostic indication.

## Data Availability Statement

The original contributions presented in the study are included in the article/Supplementary Material, further inquiries can be directed to the corresponding author.

## Author Contributions

JM, AP, VS-B, and AB designed the study. JM and OS performed the research. JM, OS, AP, and VS-B analyzed the data. JM, OS, PH, EV, AP, VS-B, and AB wrote the manuscript.

## Author Disclaimer

The views expressed in this article are the personal views of the authors and may not be understood or quoted as being made on behalf of, or reflecting the position of the EMA or one of its committees, working parties, or any of the national agencies.

## Conflict of Interest

EV initiated and led the DRUP as one of the principal investigators. The remaining authors declare that the research was conducted in the absence of any commercial or financial relationships that could be construed as a potential conflict of interest.

## Publisher's Note

All claims expressed in this article are solely those of the authors and do not necessarily represent those of their affiliated organizations, or those of the publisher, the editors and the reviewers. Any product that may be evaluated in this article, or claim that may be made by its manufacturer, is not guaranteed or endorsed by the publisher.
