# Supplementary material for: A Comparison of Post-marketing Measures Imposed by Regulatory Agencies to Confirm the Tissue-Agnostic Approach
Source: Front Med (Lausanne). 2022 Jun 14;9:893400. doi: 10.3389/fmed.2022.893400 (PMC9237332; doi:10.3389/fmed.2022.893400)
Supplement: Supplementary file 1 [file Table_1.DOCX]

**Supplementary Table 1. Distribution of tumor types within the initial datasets per medicinal product and agency**

| **product** | **Cancer** | **FDA** | **PMDA** | **EMA** |
| --- | --- | --- | --- | --- |
|  |  | **Number of patients** | | |
| Pembrolizumab | Colorectal cancer | 90 | 61 | N.A. |
|  | Endometrial cancer | 14 | 24 | N.A. |
|  | Biliary cancer | 11 | 9 | N.A. |
|  | Small intestinal cancer | 8 | 13 | N.A. |
|  | Gastric cancer | 8 | 13 | N.A. |
|  | Pancreatic cancer | 6 | 10 | N.A. |
|  | Esopageal cancer | 1 | - | N.A. |
|  | GE junction cancer | 1 | - | N.A. |
|  | Breast cancer | 2 | - | N.A. |
|  | Prostate cancer | 2 | 1 | N.A. |
|  | Bladder cancer | 1 | 2 | N.A. |
|  | Sarcoma | 1 | 1 | N.A. |
|  | Thyroid cancer | 1 | 2 | N.A. |
|  | Retroperitoneal cancer | 1 | 1 | N.A. |
|  | Small cell lung cancer | 1 | 3 | N.A. |
|  | Renal cell cancer | 1 | - | N.A. |
|  | Adrenocortical carcinoma | - | 3 | N.A. |
|  | Mesothelioma | - | 3 | N.A. |
|  | Cervical cancer | - | 2 | N.A. |
|  | Neuroendocrine tumors | - | 2 | N.A. |
|  | Brain tumor | - | 1 | N.A. |
|  | Ovarian cancer | - | 1 | N.A. |
|  | Salivary gland cancer | - | 1 | N.A. |
|  | Testicular tumor | - | 1 | N.A. |
|  | Tonsil cancer | - | 1 | N.A. |
| Larotrectinb | Soft tissue sarcoma | 11 | N.A. | 11* |
|  | Salivary gland | 12 | N.A. | 12* |
|  | Lung cancer | 4 | N.A. | 4* |
|  | Colorectal cancer | 4 | N.A. | 4* |
|  | Infantile fibrosarcoma | 7 | N.A. | 7* |
|  | Thyroid cancer | 5 | N.A. | 5* |
|  | Melanoma | 4 | N.A. | 4* |
|  | Breast cancer | 1 | N.A. | 1* |
|  | GIST | 3 | N.A. | 3* |
|  | Pancreatic cancer | 1 | N.A. | 1* |
|  | Cholangiocarcinoma | 2 | N.A. | 2* |
|  | Appendix cancer | 1 | N.A. | 1* |
| Entrectinib | Sarcoma | 13 | 13 | 13** |
|  | Non‐small cell lung cancer | 10 | 9 | 10** |
|  | Mammary analogue secretory carcinoma | 7 | 6 | 7** |
|  | Breast cancer | 6 | 6 | 6** |
|  | Thyroid cancer | 5 | 5 | 5** |
|  | Colorectal cancer | 4 | 3 | 4** |
|  | Neuroendocrine cancers | 3 | 3 | 3** |
|  | Pancreatic cancer | 3 | 3 | 3** |
|  | Gynecological cancers | 2 | 2 | 2** |
|  | Cholangiocarcinoma | 1 | 1 | 1** |

*The EMA focused its review on an extended primary analysis set, i.e. the ePAS2, but the initial primary analysis set for efficacy consisted of the first 55 patients enrolled in study LOXO-TRK-14001, LOXO-TRK-15002, and LOXO-TRK-15003. It is therefore likely that the distribution of tumor types was similar between the FDA and EMA submissions.

**The EMA focused its review on an extended primary analysis set, i.e. the ePAS, but the initial primary analysis set for efficacy consisted of the first 54 patients enrolled in ALKA‐372‐001, RXDX‐101‐01, and RXDX‐101‐02. It is therefore likely that the distribution of tumor types was similar between the FDA and EMA submissions.
