# Supplementary material for: A Comparison of Post-marketing Measures Imposed by Regulatory Agencies to Confirm the Tissue-Agnostic Approach
Source: Front Med (Lausanne). 2022 Jun 14;9:893400. doi: 10.3389/fmed.2022.893400 (PMC9237332; doi:10.3389/fmed.2022.893400)
Supplement: Supplementary file 2 [file Table_2.docx]

**Supplementary Table 2. All post-marketing measures imposed by each agency per medicinal product (source: review reports)**

| **Drug** | **Agency** | **Type of measures** | **Description of measure** |  |
| --- | --- | --- | --- | --- |
| **Pembrolizumab** | FDA | Accelerated approval requirements | Submit the final report, including datasets, from trials conducted to verify and describe the clinical benefit of pembrolizumab 200 mg intravenously every three weeks in patients with microsatellite instability high or mismatch repair deficient tumors including at least 124 patients with colorectal cancer enrolled in Merck-initiated trials; at least 300 patients with non-colorectal cancer, including a sufficient number of patients with prostate cancer, thyroid cancer, small cell lung cancer; and ovarian cancer; and 25 children. In order to characterize response rate and duration, patients will be followed for at least 12 months from the onset of response. |  |
|  |  | Postmarketing requirements under 505(o) | Conduct a trial that will characterize the safety of pembrolizumab administered intravenously at 2 mg/kg up to a maximum of 200 mg intravenously every three weeks or to determine a reasonably safe dosage regimen in an adequate number of children with primary central nervous system malignancies that are mismatch repair deficient or microsatellite instability high. Submit a final report and datasets for pediatric patients with primary CNS malignancies. |  |
|  |  | Postmarketing commitments subject to reporting requirements under section 506B | Commitment to support the availability through an appropriate analytical and clinical validation study using clinical trial data that will support labeling of an immunohistochemistry based in vitro diagnostic device that is essential to the safe and effective use of pembrolizumab for patients with tumors that are mismatch repair deficient. |  |
|  |  |  | Commitment to support the availability through an appropriate analytical and clinical validation study using clinical trial data that will support labeling of a nucleic acid-based in vitro diagnostic device that is essential to the safe and effective use of pembrolizumab for patients with tumors that are microsatellite instability high. |  |
|  | EMA | Not applicable | Not applicable |  |
|  | PMDA | Post marketing surveillance | To conduct a use-results survey after the market launch to collect information on the characteristics of patients treated with the product, to promptly collect data on the efficacy and safety of the product, and to take necessary actions for the proper use of the product. |  |
| **Larotrectinib** | **FDA** | Accelerated approval requirements | Submit the final report, including datasets, from ongoing and proposed trials conducted to verify and describe the clinical benefit of larotrectinib, through more precise estimation of the overall response rate and mature response duration per independent review assessment, in adult and pediatric patients with solid tumors with a neurotrophic receptor tyrosine kinase (NTRK) gene fusion and without a known acquired resistance mutation; are metastatic or where surgical resection is likely to result in severe morbidity; and have no satisfactory alternative treatment or that have progressed following treatment. A sufficient number of patients will be evaluated to characterize response and durability of response for each of the following tumor types: colorectal cancer, non-small cell lung cancer, central nervous system tumors, and melanoma. A minimum of 40 patients with cancers other than colorectal cancer, non-small cell lung cancer, central nervous system tumors, melanoma, soft tissue sarcoma, thyroid cancer, infantile fibrosarcoma, and salivary cancers (e.g., breast cancer, gastrointestinal stromal tumors, cholangiocarcinoma, biliary tract cancers) will also be studied. Overall response rate and duration of response will be assessed by independent central review and all responding patients will be followed for at least 12 months from the onset of response. |  |
|  |  |  | Submit the final report, including datasets, from the first 55 patients with NTRK fusion solid tumors enrolled across Study LOXO-TRK-14001 (NCT02122913), SCOUT (NCT02637687), and NAVIGATE (NCT02576431), to further characterize the duration of response in patients who achieved a complete or partial response to larotrectinib. All responding patients will be followed for at least 2 years from the onset of response and duration of response will be assessed by independent central review. |  |
|  |  | Postmarketing Requirements under 505 (o) | Conduct a study of larotrectinib in a sufficient number of pediatric patients with NTRK-fusion solid tumors to evaluate the potential serious risk of adverse longterm effects of larotrectinib on the growth and development of pediatric patients. Patients will be evaluated for growth and developmental milestones using ageappropriate screening tools and undergo neurological examination at appropriate intervals (for example, every six months) until larotrectinib is discontinued or for minimum of five years, whichever occurs first. Evaluations should include a neurologic exam, developmental milestone assessment, Karnofsky/Lansky score, growth as measured by weight and height, height velocity, height standard deviation scores (SDS), age at adrenarche if applicable (males), age at menarche if applicable (females), and Tanner Stage. |  |
|  |  |  | Conduct a study of larotrectinib 100 mg orally once daily in a sufficient number of adult or pediatric patients with a body surface area of at least 1.0 m2 who experienced an adverse reaction requiring a third dosage modification of larotrectinib to better characterize the tolerability of this approved dosage modification for larotrectinib. The following information will be provided for each patient: patient age and body surface area (if pediatric), adverse reactions leading to each prior dose reduction of larotrectinib, duration of treatment on prior dose levels, duration of treatment at the 100 mg orally once daily regimen, best overall response and duration of response. and tumor information collected while receiving the 100 mg orally once daily dosage regimen. |  |
|  |  |  | Conduct a physiologically-based pharmacokinetic modeling study to evaluate the effect of repeat doses of a moderate CYP3A4 inhibitor on the single dose pharmacokinetics of larotrectinib to address the potential for excessive drug toxicity. |  |
|  |  | Postmarketing commitments subject to reporting requirements under section 506B | Conduct a physiologically-based pharmacokinetic modeling study to evaluate the effect of repeat doses of a moderate CYP3A4 inducer on the single dose pharmacokinetics of larotrectinib to assess the magnitude of decreased drug exposure and to determine appropriate dosing recommendations. |  |
|  |  |  | Conduct an analytical and clinical validation study, using clinical trial data, that is adequate to support labeling of an in vitro diagnostic device that is essential to the safe and effective use of larotrectinib for patients with NTRK gene fusions in solid tumor specimens. |  |
|  | **EMA** | Specific Obligations | In order to further confirm the histology-independent efficacy of larotrectinib and to investigate the primary and secondary resistance mechanisms, the MAH should submit a pooled analysis for the increased sample size including the final report of study LOXO-TRK-15002 (NAVIGATE). |  |
|  |  |  | In order to further investigate the long-term toxicity and developmental effects of larotrectinib in paediatric patients, with particular focus on neurodevelopment including cognitive function, the MAH should submit the final report of study LOXO-TRK-15003 (SCOUT) including 5 year follow up data. |  |
|  |  |  | In order to further confirm the appropriate dose recommended in paediatric patients, the MAH should submit an updated pop PK model based on additional PK sampling in patients aged 1 month to 6 years from study LOXO-TRK-15003 (SCOUT). |  |
|  | **PMDA** | Not Applicable | Not applicable |  |
| **Entrectinib** | **FDA** | Accelerated approval requirements | Submit the final report, including datasets, from the first 54 patients with NTRK-fusion solid tumors enrolled across the ALKA, STARTRK-1 [NCT02097810], and STARTRK-2 [NCT02568267] studies to verify and describe the clinical benefit and further characterize the duration of response in patients who achieved a complete or partial response to entrectinib. All responding patients will be followed for at least 2 years from the onset of response or until disease progression, whichever comes first. Duration of response will be assessed by independent central review. |  |
|  |  |  | Submit the final report, including datasets, from ongoing and proposed trials conducted to verify and describe the clinical benefit of entrectinib, through more precise estimation of the overall response rate and mature response duration per independent review assessment, in adult and pediatric patients 12 years of age and older with solid tumors with a neurotrophic receptor tyrosine kinase (NTRK) gene fusion and without a known acquired resistance mutation; are metastatic or would require surgical resection that would result in severe morbidity; and have no satisfactory alternative treatment or that have progressed following treatment.   A sufficient number of patients will be evaluated to more precisely characterize response and durability of response for each of the following tumor types: pediatric solid tumors, colorectal cancer, central nervous system cancers, gynecological cancers, and melanoma.   A minimum of 40 patients with cancers other than pediatric solid tumors, colorectal cancer, central nervous system cancers, gynecological cancers, melanoma, soft tissue sarcoma, non-small cell adenocarcinoma lung cancer, mammary analogue secretory carcinoma, and secretory breast cancer will also be studied. Overall response rate and duration of response will be assessed by independent central review and all responding patients will be followed for at least 12 months from the onset of response. |  |
|  |  | Postmarketing requirments under 505(o) | Determine functional activation or inhibition of off-target receptors, transporters, and/or channels that, at concentrations of 10 µM, showed greater than 50% inhibition by entrectinib or M5 in the secondary pharmacology studies submitted to NDA 212725 and 212726. As part of an integral safety assessment, include EC50 or IC50 data for target receptors, transporters, and channels that are still significantly affected at a concentration less than 1 µM, particularly those involved in suicidal intent and behavior, as described in Muller et al., 2015. |  |
|  |  |  | Submit integrated safety analyses and supporting data from an adequate number of patients enrolled in clinical trial(s) designed to characterize the cardiac risks and its sequelae in patients exposed to entrectenib with reasonable precision; to identify risk factors for development of these sequelae; and to support labeling instructions for dose modification and monitoring. The design of the trial should include sufficient cardiac monitoring to achieve these objectives. |  |
|  |  |  | Conduct clinical trial(s) of entrectinib in a sufficient number of pediatric patients 12 years of age and older with NTRK-fusion solid tumors to evaluate the potential serious risk of adverse long-term effects of entrectinib on growth and development, including neurological outcomes with reasonable precision. Patients will be monitored for growth and developmental milestones using age-appropriate screening tools and undergo neurological examination at appropriate intervals. Evaluations will include neurological exams with neurocognitive assessment, Karnofsky/Lansky score, growth as measured by height, weight, height velocity, and height standard deviation scores (SDS), age at adrenarche if applicable (males), age at menarche if applicable (females) and Tanner Stage. Patient monitoring will be performed until discontinuation of study treatment or a minimum of 5 years from start of treatment, whichever occurs first. |  |
|  |  |  | Submit integrated safety analyses and supporting data from an adequate number of patients enrolled in clinical trial(s) designed to characterize the risk of fractures and its sequelae in patients exposed to entrectinib with reasonable precision; to identify risk factors for development of these sequelae; and to support labeling recommendations to mitigate the risk of skeletal fractures. The design of the trial should include sufficient bone monitoring to achieve these objectives, including but not limited to initial and serial assessment of bone mineral density (BMD) with dual x-ray absorptiometry (DXA) scans, and markers of bone formation, bone resorption, and calcium metabolism. |  |
|  |  |  | Complete a pharmacokinetic trial to evaluate the effect of moderate and severe hepatic impairment on the pharmacokinetics and safety of Rozlytrek (entrectinib) compared to subjects with normal hepatic function in accordance with the FDA Guidance for Industry entitled, “Pharmacokinetics in Patients with Impaired Hepatic Function: Study Design, Data Analysis, and Impact on Dosing and Labeling,” available at: https://www.fda.gov/media/71311/download. |  |
|  |  | Postmarketing commitments subject to reporting requirements under section 506B | Commit to providing adequate analytical and clinical validation results from clinical trial data to support labeling of the F1CDx test to detect NTRK rearrangements for identifying patients who may benefit from entrectinib. The analytical validation should consist of precision, limit of detection, and accuracy studies for the NTRK indication. The clinical validation should be supported by a clinical bridging study comparing F1CDx and the clinical trial enrollment assays. |  |
|  | **EMA** | Specific Obligation | In order to further confirm the histology-independent efficacy of entrectinib in adult and paediatric patients, the MAH should submit a pooled analysis for an increased sample size of NTRK fusion-positive patients from the ongoing studies STARTRK-2, STARTRK-NG and any additional clinical trial conducted according to an agreed protocol. The MAH should submit the results of an interim safety and efficacy analysis of the NTRK efficacy-evaluable adult and paediatric patients including adolescents that are available as per integrated statistical analysis plan. |  |
|  |  |  | In order to further investigate the impact of the presence/absence of other molecular alteration on the efficacy of entrectinib, the MAH should submit the results from tumour genomic profiling by plasma and/or tissue when possible at baseline and progression together with clinical outcomes association per tumour histology for the patients from the updated pooled analysis. |  |
|  | **PMDA** | post-marketing surveillance | to conduct a post-marketing use-results survey covering all patients treated with the product, until data from a certain number of patients are collected, in order to obtain information on the characteristics of patients treated with the product, to promptly collect data on the safety and efficacy of the product, and to take necessary measures to ensure proper use of the product. |  |
|  | **PMDA** | post-marketing surveillance | To conduct a post-marketing use-results survey to investigate delayed growth and development in pediatric patients in clinical practice. |  |
